# Road Traffic Noise and Incidence of Primary Hypertension: A Prospective Analysis in UK Biobank

**DOI:** 10.1016/j.jacadv.2023.100262

**Published:** 2023-03-22

**Authors:** Jing Huang, Teng Yang, John Gulliver, Anna L. Hansell, Mohammad Mamouei, Yutong Samuel Cai, Kazem Rahimi

**Affiliations:** a Department of Occupational and Environmental Health Sciences, School of Public Health, Peking University, Beijing, China; b Deep Medicine, Nuffield Department of Women’s and Reproductive Health, University of Oxford, Oxford, United Kingdom; c Centre for Environmental Health and Sustainability, University of Leicester, Leicester, United Kingdom; d National Institute for Health Research Health Protection Research Unit in Environmental Exposures and Health at the University of Leicester, Leicester, United Kingdom

**Keywords:** air pollution, long-term exposure, primary hypertension, prospective study, road traffic noise

## Abstract

**Background:**

The quality of evidence regarding the associations between road traffic noise and hypertension is low due to the limitations of cross-sectional study design, and the role of air pollution remains to be further clarified.

**Objectives:**

The purpose of this study was to evaluate the associations of long-term road traffic noise exposure with incident primary hypertension; we conducted a prospective population-based analysis in UK Biobank.

**Methods:**

Road traffic noise was estimated at baseline residential address using the common noise assessment method model. Incident hypertension was ascertained through linkage with medical records. Cox proportional hazard models were used to estimate hazard ratios (HRs) for association in an analytical sample size of over 240,000 participants free of hypertension at baseline, adjusting for covariates determined via directed acyclic graph.

**Results:**

During a median of 8.1 years follow-up, 21,140 incident primary hypertension (International Classification of Diseases-10th Revision [ICD-10]: I10) were ascertained. The HR for a 10 dB[A] increment in mean weighted average 24-hour road traffic noise level (*L*_*den*_) exposure was 1.07 (95% CI: 1.02-1.13). A dose-response relationship was found, with HR of 1.13 (95% CI: 1.03-1.25) for *L*_*den*_ >65 dB[A] vs ≤55 dB[A] (*P* for trend <0.05). The associations were all robust to adjustment for fine particles (PM_2.5_) and nitrogen dioxide (NO_2_). Furthermore, high exposure to both road traffic noise and air pollution was associated with the highest hypertension risk.

**Conclusions:**

Long-term exposure to road traffic noise was associated with increased incidence of primary hypertension, and the effect estimates were stronger in presence of higher air pollution.

Elevated blood pressure contributes more to cardiovascular disease (CVD) and premature death than any other known and modifiable risk factor.^1^ It was estimated that over 30% of adults (1.39 billion) worldwide had hypertension in 2010 (defined as systolic blood pressure ≥140 mmHg and/or diastolic blood pressure ≥90 mmHg)^1^; and as the population is ageing, the prevalence is expected to rise, which, if left untreated, would cause tremendous disease burden.^2^ To mitigate the growing burden of hypertension, identification of its modifiable risk factors at both individual and population levels remains an instrumental strategy. The 2021 European Society of Cardiology Guidelines on CVD prevention for the first time highlighted environmental exposures including air pollution and above-threshold noise levels as having CVD risk modifying potential.^3^

Traffic noise has emerged as an important environmental risk factor for CVD since 2010, with road traffic noise being mostly investigated for the associated health effects.^4^ Epidemiological and animal studies indicate that traffic noise can trigger annoyance and disturb sleep, thus activating autonomic system and overproducing stress hormones, with subsequent activation of the renin-angiotensin-aldosterone system.^5^ Chronic exposure to traffic noise may cause a number of pathophysiological adaptations, such as increase in heart rate and cardiac output and a rise in blood pressure. It could also lead to development of other cardiovascular risk factors such as hyperglycemia, hypercholesterolemia, and blood clotting factor activation. These changes would ultimately manifest as CVD.^4^^,^^6^, ^7^, ^8^

Despite the biological plausibility of the link between chronic exposure to traffic noise and the risk of hypertension, epidemiological studies exploring long-term exposure to road traffic noise and prevalent or incident hypertension indicated inconsistent results. For instance, a 2018 World Health Organization (WHO) meta-analysis of 26 cross-sectional studies published between 2000 and 2014 indicated a relative risk of 1.05 (95% CI: 1.02-1.08) per 10 dB for the association between road traffic noise and hypertension prevalence, with the evidence quality of *very low* due to the nature of cross-sectional study design.^9^ An updated meta-analysis based on 14 cohort and case-control studies published between 2011 and 2017 reported that the incidence risk of hypertension was 1.02 (95% CI: 0.98-1.05) per 10 dB increase in road traffic noise. The quality of evidence was rated as *low*, indicating that high quality prospective studies are needed to further strengthen this risk estimate.^10^

The impact of ambient air pollution on hypertension is relatively well documented, although not all previous studies have considered both air pollution and road traffic noise contemporaneously. Given that road traffic commonly leads to both noise and air pollution,^11^^,^^12^ it is important to address the potential interaction or confounding between these 2 common environmental exposures in relation to any CVD outcomes.

We recently reported in a cross-sectional analysis that long-term exposure to road traffic noise above 65 dB[A] was associated with small but statistically significant elevations in both systolic and diastolic blood pressure in the UK Biobank cohort, independent of air pollution.^13^ Based on this previous work, we further conducted a prospective analysis by investigating the association between long-term residential road traffic noise and incidence of primary hypertension in this large cohort. Furthermore, stratified analyses were performed to identify potentially susceptible populations, and the influence of both particulate and gaseous air pollution on hypertension was considered to disentangle that from road traffic noise.

## Methods

### Study design and population

UK Biobank is a large cohort study.^14^ In brief, over 500,000 participants aged 40 to 69 years were enrolled from 22 study assessment centers across the UK from 2006 to 2010. A wide range of sociodemographic and health-related information was collected through touchscreen questionnaires, physical and biological measurements at baseline, with follow-up of disease morbidity and mortality via record linkages. The UK Biobank study was approved by the North West Multi-Centre Research Ethical Committee, and written consent was provided by all participants.

We conducted a prospective study, in which participants with hypertension at baseline (identified either through the first occurrence medical records or self-reported answers via verbal interview) were excluded from the analysis. The remaining participants were then followed up to March 31, 2017, for the first occurrence of primary hypertension.

### Primary hypertension ascertainment

The outcome of incident primary hypertension was extracted from the “first reported primary hypertension.” The 10th Revision code of a 3-character International Statistical Classification of Diseases and Related Health Problems was used to identify primary hypertension (International Classification of Diseases-10th Revision [ICD-10]: I10). The diagnosis of primary hypertension was ascertained by hospital admissions, primary care, and self-reported.

### Exposure assessment

The common noise assessment method- European Union (CNOSOS-EU) model was used to estimate the residential address-based annual mean road traffic noise level. This model has been formulated as a strategic noise mapping tool which provides adequate performance for exposure ranking (Spearman’s rank: 0.75),^15^ and it was also validated for epidemiological studies.^13^^,^^16^ Detailed information on road traffic flow, vehicle classification, point source representation of a vehicle, and vehicle sound power emission was integrated in the road-traffic noise model,^17^ as well as parameters such as noise propagation, meteorological conditions, land over, building heights, and topography.^15^ Annual mean A-weighted sounds pressure level in decibels (dB[A]) for 2009 was assessed based on all road sources within 500 m of residential address in the study. Two indicators of road traffic noise exposure were considered: *L*_*den*_ (weighted average 24-hour road traffic noise level, with a 5 and 10 dB penalty added to the evening and night levels, respectively) and *L*_*night*_ (average night-time road traffic noise level from 23:00 to 7:00).

The air pollution levels were linked to participants’ residential address at the baseline. Annual air pollution levels including fine particles (PM_2.5_) and nitrogen dioxide (NO_2_) concentrations at baseline address were estimated by land use regression models, with the model performance for annual PM_2.5_ and NO_2_ in 2010 higher than 80%.^18^^,^^19^

### Covariates

Demographic characteristics including age, sex, ethnic background, as well as socioeconomic status indicators including education, economic status, average total household income before tax, and Townsend area-level deprivation index were included in the models as covariates. Lifestyle factors including smoking status, alcohol intake frequency, salt intake, physical activity level, sedentary time, and sleep duration were also considered. Serum concentrations of glycated hemoglobin (mmol/mol), high density lipoprotein cholesterol (mmol/L), and triglycerides (mmol/L) were obtained from blood assays. Central obesity status was derived using European waist circumference cut-off value of 80 and 94 cm for female and male, respectively.^20^ Assessment center and length of time at residence at the time of baseline recruitment were also considered. Furthermore, hearing loss information was also obtained.

### Statistical analysis

Cause-specific hazard models which took competing risks into consideration were used to estimate the hazard ratios (HRs) for the association between road traffic noise and incidence of primary hypertension.^21^^,^^22^ We used Schoenfeld residuals to test the proportional hazards assumption. Follow-up time was calculated from the recruitment date to the first diagnosis of primary hypertension, lost to follow-up, death, or end of the current follow-up, whichever came first.^23^

Associations were analyzed between both continuous road traffic noise (expressed per 10 dB[A]) and categories of road traffic noise and incident primary hypertension adjusting for the covariates. *L*_*den*_ was categorized as ≤55, >55 to ≤60, >60 to ≤65, and >65 dB[A] and *L*_*night*_ was categorized as ≤45, >45 to ≤50, >50 to ≤55, and >55 dB[A], with reference value of ≤55 and ≤45 dB[A] for *L*_*den*_ and *L*_*night*_, respectively.^13^ Nested models were constructed as follows: model 1, fully adjusted model including adjustment for age, sex, ethnicity, education, economic status, average total household income before tax, Townsend deprivation index, assessment center, length of time at residence, glycated hemoglobin, high density lipoprotein cholesterol, triglycerides, smoking status, alcohol intake frequency, salt intake and hearing loss; model 2, with additional adjustment for PM_2.5_; and model 3, with additional adjustment for NO_2_.

Covariates for the statistical models were selected based on existing literature, biological plausibility, and availability of data as shown in the directed acyclic graph (Supplemental Figure 1).

The exposure-response relationship between road traffic noise and incident primary hypertension was explored by using a restricted cubic spline analysis with 3 knots in the continuous noise exposure models.

The associations between air pollution and incidence of primary hypertension were explored by adjusting for the covariates in the model 1, and plus adjusted for traffic noise. Furthermore, to explore the combined effects of traffic noise and air pollution, we generated 4 stratified traffic noise levels with 3 stratified air pollution levels into 12 categories. Participants were stratified into residing in 3 air pollution areas according to ≤*P*_*50*_, *P*_*50*_ and *P*_*75*_, and > *P*_*75*_ of the air pollution concentrations. We were unable to categorize low and high air pollution areas based on the recently updated WHO air quality guideline on annual mean PM_2.5_ (5 μg/m^3^) and NO_2_ (10 μg/m^3^), as none of our participants had an annual mean for either pollutants below the guideline value. The effect of high exposure to both traffic noise and air pollution on risk for primary hypertension were investigated, with the category of low traffic noise and low air pollution used as the reference group (categorical analysis).

Possible effect modifications by age (<65 years, ≥65 years), sex (female, male), economic status (inactive, active), Townsend deprivation index (from least deprived to most deprived), length of time at residence (<10 years, ≥10 years), salt intake (never/rarely, sometimes, usually, and always), sedentary time (≤4.5 hours, >4.5 hours), and physical activity level (low, moderate, and high) were explored. The homogeneity across stratum-specific HRs was tested using the interaction between categorized road traffic noise and each potential modifier.

Sensitivity analyses were conducted to confirm the robustness of the results: 1) further adjustment for physical activity level and central obesity status; 2) further adjustment for sedentary time and sleep duration; and 3) excluding those who were identified with primary hypertension within 2 years during the follow-up period considering the potential time window between road traffic noise exposure and primary hypertension occurrence, providing evidence against reverse causation.^24^

All analyses were performed using R software (version 3.6.3), and 2-sided *P* value <0.05 was considered as statistically significant.

## Results

The mean age of the study participants at recruitment was 55.0 years, and 54.6% were female (Table 1). The mean residential *L*_*den*_ was 56.0 dB[A], with a range from 51.5 dB[A] to 87.0 dB[A]. Those exposed to *L*_*den*_ >55 dB[A] and *L*_*night*_ >45 dB[A] accounted for 48.0% and 58.5% of the study participants, respectively. The annual mean PM_2.5_ and NO_2_ concentrations were 10.0 μg/m^3^ and 26.5 μg/m^3^, respectively. The Spearman correlations between *L*_*den*_ with PM_2.5_ and NO_2_ were 0.24 and 0.23, respectively. There were high correlations between PM_2.5_ and NO_2_, and between *L*_*den*_ and *L*_*night*_ (Supplemental Table 1).

**Table 1:** Baseline Characteristics and Environmental Exposures of Study Participants (N = 246,447)

Sex (n = 246,447)	
Female	134,439 (54.6%)
Male	112,008 (45.4%)
Age at baseline (y) (n = 246,447)	55.0 ± 8.1
Ethnicity (n = 246,447)	
White	236,151 (95.8%)
Other	10,296 (4.2%)
Education (n = 246,447)	
College or university degree	92,085 (37.4%)
A levels/AS levels or equivalent	29,996 (12.2%)
O levels/GCSEs or equivalent	52,805 (21.4%)
CSEs or equivalent	13,987 (5.7%)
NVQ or HND or HNC or equivalent	15,181 (6.2%)
Other professional qualifications	11,811 (4.8%)
None of the above	30,582 (12.4%)
Economic status (n = 246,447)	
Active	162,149 (65.8%)
Inactive	84,298 (34.2%)
Average total household income before tax (n = 246,447)	
<£18,000	47,971 (19.5%)
£18,000-£30,999	60,104 (24.4%)
£31,000-£51,999	67,468 (27.4%)
£52,000-£100,000	55,727 (22.6%)
>£100,000	15,177 (6.2%)
Townsend deprivation index (n = 246,447)	
1 (least deprived)	49,338 (20.0%)
2	49,255 (20.0%)
3	49,285 (20.0%)
4	49,280 (20.0%)
5 (most deprived)	49,289 (20.0%)
Length of time at residence (n = 246,447)	
<10 y	83,985 (34.1%)
≥10 y	162,462 (65.9%)
HbA1c (mmol/mol) (n = 246,447)	35.2 ± 5.6
HDL cholesterol (mmol/L) (n = 246,447)	1.5 ± 0.4
Triglycerides (mmol/L) (n = 246,447)	1.4 ± 1.1
Smoking status (n = 246,447)	
Never smoker	138,509 (56.2%)
Former smoker	81,176 (32.9%)
Current smoker	26,762 (10.9%)
Alcohol intake frequency (n = 246,447)	
Never	16,403 (6.7%)
Special occasions only	25,053 (10.2%)
1-3 times a month	27,886 (11.3%)
Once or twice a week	65,174 (26.4%)
3 or 4 times a week	60,799 (24.7%)
Daily or almost daily	51,132 (20.7%)
Salt intake (n = 246,447)	
Never/rarely	135,382 (54.9%)
Sometimes	70,077 (28.4%)
Usually	29,426 (11.9%)
Always	11,562 (4.7%)
Sedentary time (h) (n = 241,511)	4.7 ± 2.3
Sleep duration (h) (n = 245,722)	7.2 ± 1.0
Physical activity level (n = 208,254)	
Low	37,445 (18.0%)
Moderate	84,946 (40.8%)
High	85,863 (41.2%)
Central obesity status (n = 246,055)	
Yes	133,409 (54.2%)
No	112,646 (45.8%)
Hearing loss (n = 246,447)	
Yes	8,239 (3.3%)
No	238,208 (96.7%)
*L*_*den*_ (dB[A]) (n = 246,447)	56.0 ± 4.2 (51.5-87.0)
Low (≤55 dB[A])	128,142 (52.0%)
Low-medium (>55-≤60 dB[A])	88,577 (35.9%)
Medium-high (>60-≤65 dB[A])	14,450 (5.9%)
High (>65 dB[A])	15,278 (6.2%)
*L*_*night*_ (dB[A]) (n = 246,447)	46.6 ± 4.2 (42.1-77.6)
Low (≤45 dB[A])	102,238 (41.5%)
Low-medium (>45-≤50 dB[A])	111,159 (45.1%)
Medium-high (>50-≤55 dB[A])	16,668 (6.8%)
High (>55 dB[A])	16,382 (6.6%)
PM_2.5_ (μg/m^3^) (n = 228,284)	10.0 ± 1.1 (8.2-21.3)
NO_2_ (μg/m^3^) (n = 246,447)	26.5 ± 7.6 (12.9-107.8)

Values are n (%), mean ± SD, or mean ± SD (range). Participants with hypertension at baseline identified either through first occurrence medical records, or self-reported answers via verbal interview were excluded.

*AS* = Advanced Subsidiary; GCSE = *General Certificate of Secondary Education;* HbA1c = glycated hemoglobin; HDL = high density lipoprotein; HNC = Higher National Certificate; HND = Higher National Diploma; IQR = interquartile range; *L*_*den*_ = weighted average 24-h road traffic noise level; *L*_*night*_ = average night-time road traffic noise level from 23:00 to 7:00; NO_2_ = nitrogen dioxide; NVQ = National Vocational Qualification; PM_2.5_ = fine particles; SD = standard deviation.

During a median of 8.1 years follow-up (a total of 1,920,332 person years), 21,140 incident primary hypertension cases were identified from 246,447 participants without hypertension at baseline. Road traffic noise exposure at the residence was associated with a higher risk of incident primary hypertension. In the fully adjusted model (model 1), the associations between categories of road traffic noise and incident primary hypertension showed a dose-response relationship, with HRs of 1.13 [95% CI: 1.03-1.25] for *L*_*den*_ >65 dB[A] vs ≤55 dB[A] (*P* for trend <0.05), and HRs of 1.13 (95% CI: 1.03, 1.24) for *L*_*night*_ >55 dB[A] vs ≤45 dB[A] (*P* for trend <0.05) (Table 2). The associations were all robust to adjustment for PM_2.5_ and NO_2_: The HRs were 1.13 (95% CI: 1.02-1.25) and 1.11 (95% CI: 1.00-1.23) for *L*_*den*_ >65 dB[A] vs ≤55 dB[A] when adjusting for PM_2.5_ and NO_2_, respectively; and the HRs were 1.13 (95% CI: 1.02-1.25) and 1.10 (95% CI: 1.00-1.22) for *L*_*night*_ >55 dB[A] vs ≤45 dB[A] when adjusting for PM_2.5_ and NO_2_, respectively (Table 2).

**Table 2:** The Associations Between Exposure to Road Traffic Noise and Incidence of Primary Hypertension

Road Traffic Noise	Number of Cases	Model 1, HR (95% CI)	Model 2, HR (95% CI)	Model 3, HR (95% CI)
*L*_*den*_				
Low (≤55 dB[A])	10,978	ref	ref	ref
Low-medium (>55-≤60 dB[A])	7,540	1.02 (0.98–1.07)	1.02 (0.97–1.08)	1.02 (0.97–1.07)
Medium-high (>60-≤65 dB[A])	1,270	1.05 (0.96–1.16)	1.04 (0.94–1.15)	1.05 (0.96–1.16)
High (>65 dB[A])	1,352	**1.13 (1.03–1.25)**	**1.13 (1.02–1.25)**	**1.11 (1.00–1.23)**
*P* for trend		0.007	0.020	0.030
Continuous 24-h road traffic noise	21,140	**1.07 (1.02–1.13)**	**1.06 (1.00–1.12)**	**1.06 (1.00–1.12)**
*L*_*night*_				
Low (≤45 dB[A])	8,751	ref	ref	ref
Low-medium (>45-≤50 dB[A])	9,460	1.01 (0.96–1.06)	1.01 (0.96–1.06)	1.01 (0.96–1.06)
Medium-high (>50-≤55 dB[A])	1,472	1.07 (0.98–1.17)	1.05 (0.95–1.15)	1.07 (0.98–1.17)
High (>55 dB[A])	1,457	**1.13 (1.03–1.24)**	**1.13 (1.02–1.25)**	**1.10 (1.00–1.22)**
*P* for trend		0.005	0.017	0.022
Continuous nighttime road traffic noise	21,140	**1.07 (1.02–1.13)**	**1.06 (1.00–1.12)**	**1.06 (1.00–1.12)**

Model 1: fully adjusted model. Adjusted for age, sex, ethnicity, education, economic status, average total household income before tax, Townsend deprivation index, assessment center, length of time at residence, HbA1c, HDL cholesterol, triglycerides, smoking status, alcohol intake frequency, salt intake, and hearing loss.

Model 2: fully adjusted model plus adjusted for PM_2.5_.

Model 3: fully adjusted model plus adjusted for NO_2_.

*P* for trend was based on the median value for each category. **Bold** represents significance at *P* < 0.05.

HbA1c = glycated hemoglobin; HDL = high density lipoprotein; *L*_*den*_ = weighted average 24-h road traffic noise level; *L*_*night*_ = average night-time road traffic noise level from 23:00 to 7:00.

In the fully adjusted models investigating continuous exposure, the HR was 1.07 (95% CI: 1.02-1.13) increase in incident primary hypertension per 10 dB[A] increase in *L*_*den*_. Similar trends were found for continuous *L*_*night*_ exposure. The associations were also robust when adjusting for PM_2.5_ and NO_2_ (Table 2).

**Figure 1:**
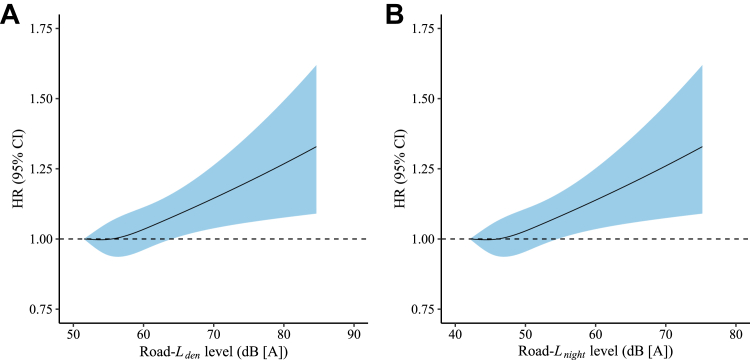
**Exposure-Response Curve Between Road Traffic Noise and Incident Primary Hypertension, The Curve was Based on the Adjusted Covariables Shown in the Fully Adjusted Model** **(A)** Weighted average 24-hour road traffic noise level (*L*_*den*_); **(B)** Average night-time road traffic noise level from 23:00 to 7:00 (*L*_*night*_). *L*_*den*_ = weighted average 24-hour road traffic noise level; *L*_*night*_ = average night-time road traffic noise level from 23:00 to 7:00.

The exposure-response curves between road traffic noise (*L*_*den*_ and *L*_*night*_) and incident primary hypertension were almost linear, with HRs increasing continuously with higher road traffic noise exposure (Figure 1).

Elevated HRs of air pollution on incident primary hypertension were also observed (Table 3). HRs were 1.17 (95% CI: 1.03-1.32) per 5 μg/m^3^ of PM_2.5_, and 1.04 (95% CI: 1.00-1.08) per 10 μg/m^3^ of NO_2_. When adjusting for traffic noise, the association for PM_2.5_ was 1.12 (95% CI: 0.99-1.28) (*P* = 0.073), and the relationship for NO_2_ reduced to 1.03, with wider CI (95% CI: 0.99-1.07) (*P* = 0.167). Table 4 shows the combined effects of traffic noise and air pollution in relation to risk of incident primary hypertension. The strongest association was found for the combination of high exposure to both road traffic noise and air pollution, where *L*_*den*_ >65 dB[A] and PM_2.5_ >10.6 μg/m^3^ were associated with HR of 1.22 (95% CI: 1.06, 1.40) compared with *L*_*den*_ ≤55 dB[A] and PM_2.5_ ≤9.9 μg/m^3^ (*P* < 0.05). Similarly, *L*_*den*_ >65 dB[A] and NO_2_ >31.1 μg/m^3^ were associated with HR of 1.18 (95% CI: 1.04-1.34) compared with *L*_*den*_ ≤55 dB[A] and NO_2_ ≤26.0 μg/m^3^ (*P* < 0.05). The same trends were seen for *L*_*night*_ (Supplemental Table 2).

**Table 3:** Association Between Air Pollution and Incidence of Primary Hypertension

Air Pollutants	Number of Cases	HR (95% CI)	*P* Value
PM_2.5_			
Not adjusted for *L*_*den*_	19,438	1.17 (1.03–1.32)	0.017
Adjusted for *L*_*den*_	19,438	1.12 (0.99–1.28)	0.073
NO_2_			
Not adjusted for *L*_*den*_	21,140	1.04 (1.00–1.08)	0.033
Adjusted for *L*_*den*_	21,140	1.03 (0.99–1.07)	0.167

The results were based on adjusting for the covariates shown in the fully adjusted model.

Results are presented per given exposure increment. The exposure increments corresponded to 5 μg/m^3^ for PM_2.5_, and 10 μg/m^3^ for NO_2_.

*L*_*den*_ = weighted average 24-h road traffic noise level; NO_2_ = nitrogen dioxide; PM_2.5_ = fine particles.

**Table 4:** Combined Effects of Weighted Average 24-hour Road Traffic Noise (*L*_*den*_) and Air Pollution on Risk of Incident Primary Hypertension

Air Pollution	The Influence of 24-h Road Traffic Noise Exposure (*L*_*den*_)			
	Low (≤55 dB[A])	Low-medium (>55-≤60 dB[A])	Medium-high (>60-≤65 dB[A])	High (>65 dB[A])
PM_2.5_				
≤9.9 μg/m^3^	ref	1.01 (0.94–1.08)	1.06 (0.91–1.23)	1.02 (0.85–1.23)
9.9-10.6 μg/m^3^	0.99 (0.92–1.06)	1.05 (0.96–1.14)	0.96 (0.77–1.20)	1.21 (0.97–1.50)
>10.6 μg/m^3^	1.04 (0.95–1.15)	1.05 (0.96–1.15)	1.09 (0.93–1.28)	**1.22 (1.06–1.40)**
NO_2_				
≤26.0 μg/m^3^	ref	1.02 (0.95–1.09)	1.04 (0.91–1.19)	1.01 (0.83–1.22)
26.0-31.1 μg/m^3^	1.02 (0.95–1.10)	1.09 (1.00–1.18)	1.03 (0.85–1.25)	1.16 (0.93–1.44)
>31.1 μg/m^3^	0.98 (0.89–1.08)	0.98 (0.90–1.08)	1.11 (0.93–1.32)	**1.18 (1.04–1.34)**

Values are HR (95% CI). PM_2.5_ and NO_2_ concentrations were divided by ≤*P*_*50*_, *P*_*50*_ and *P*_*75*_, and >*P*_*75*_.

**Bold** represents significance at *P* < 0.05.

*L*_*den*_ = weighted average 24-h road traffic noise level; NO_2_ = nitrogen dioxide; PM_2.5_ = fine particles.

We summarize in the Central Illustration the HRs for the association between road traffic noise and incident primary hypertension, and the influence of air pollution.

**Central Illustration:**
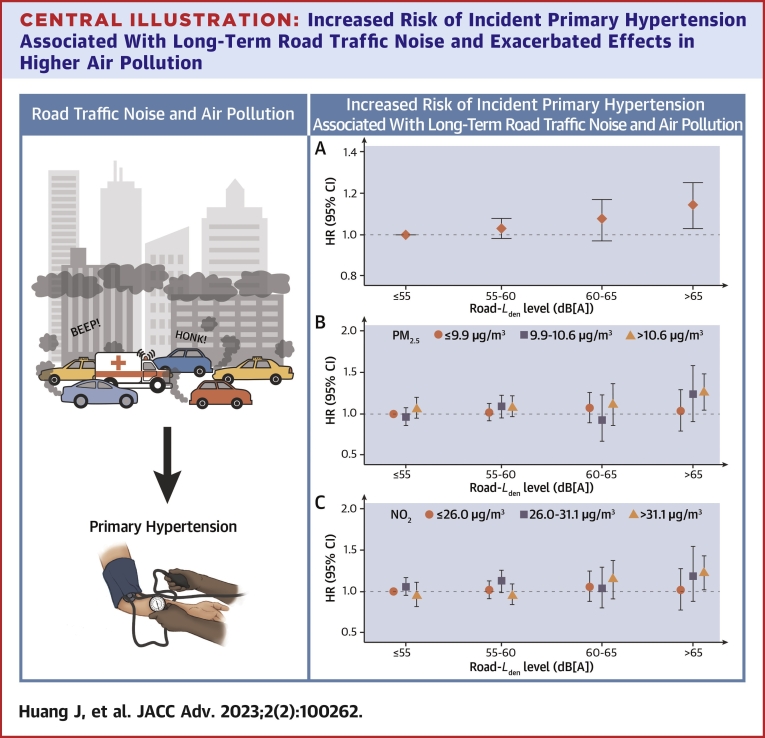
**Increased Risk of Incident Primary Hypertension Associated With Long-Term Road Traffic Noise and Exacerbated Effects in Higher Air Pollution** **(A)** The association between road traffic noise and incident primary hypertension, **(B)** The combined effects of road traffic noise and PM_2.5_ on incident primary hypertension, **(C)** The combined effects of road traffic noise and NO_2_ on incident primary hypertension. *L*_*den*_ = weighted average 24-hour road traffic noise level; *L*_*night*_ = average nighttime road traffic noise level from 23:00 to 7:00.

**Figure 2:**
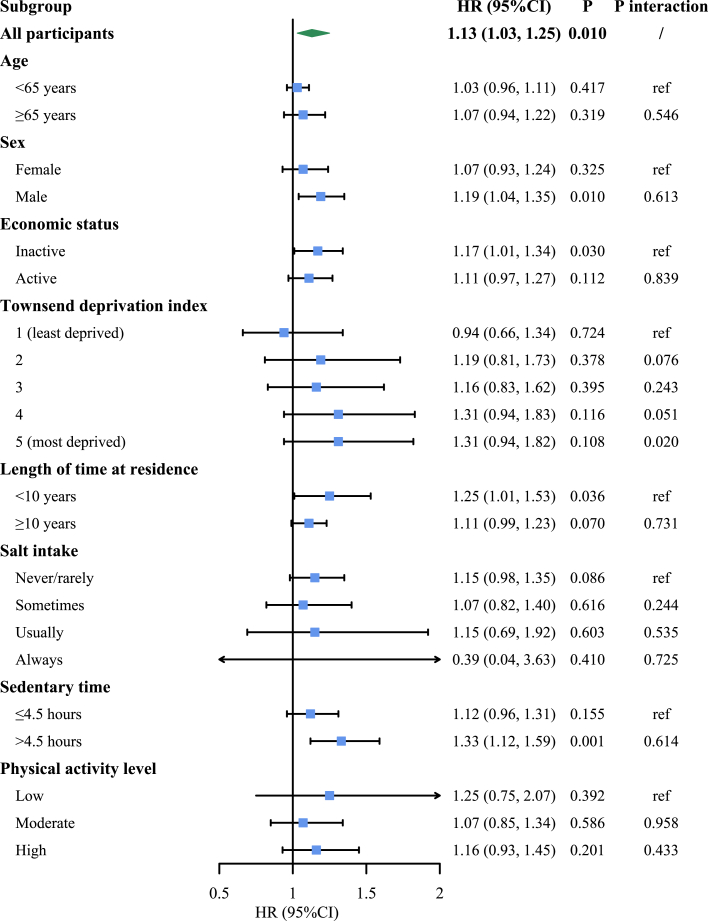
**The Association Between Road Traffic Noise Exposure and Incident Primary Hypertension Stratified by Personal Characteristics** Weighted average 24-hour road traffic noise level stratified by ≤55 dB[A], >55 to ≤60 dB[A], >60 to ≤65 dB[A], and >65 dB[A] was used. The results were based on adjusting for the covariates shown in the fully adjusted model.

For effect modification analyses, we found that the most deprived group had significantly higher risk than the least deprived group in both *L*_*den*_ >65 dB[A] vs ≤55 dB[A] and *L*_*night*_>55 dB[A] vs ≤45 dB[A] (*P* interaction <0.05) (Figure 2). Other personal characteristics did not seem to have significant modification effects and the detailed information is shown (Supplemental Tables 3 and 4).

Sensitivity analyses showed that the association between road traffic noise and incident primary hypertension was robust after further adjustment for central obesity status and physical activity level, or adjustment for sedentary time and sleep duration. After excluding those who were identified with primary hypertension within 2 years during the follow-up period, the results remained robust (Supplemental Table 5).

## Discussion

In this prospective analysis of 246,477 study participants followed for a median of 8.1 years in UK Biobank, we found that higher long-term residential road traffic noise exposure was associated with an increased risk of incident primary hypertension in a dose-response relationship. Categorical analyses suggested that associations were highest in participants with annual mean *L*_*den*_ >65 dB[A] or *L*_*night*_ >55 dB[A], and the associations were all robust to adjustment for PM_2.5_ and NO_2_. In combined effects analyses, the highest incident risk was seen in those exposed to the highest level of both road traffic noise and air pollution, suggesting effects of road traffic noise on incident primary hypertension may be exacerbated in areas where air pollution level is high.

The associations of road traffic noise exposure with hypertension have been studied previously.^8^ However, the quality of the evidence, as summarized in previous meta-analyses, has been rated as *very low* to *low* because many studies used cross-sectional or case-control design, which by nature limit interpretation on causality.^9^^,^^10^ Since 2010, prospective study designs have increasingly been utilized; however, in a 2018 review of these studies observed that there was a positive but no significantly pooled estimate.^10^ The ESCAPE (Evaluation Study of Congestive Heart Failure and Pulmonary Artery Catheterization Effectiveness) study of 7 European cohorts (none from the UK) found a weak association (HR: 1.03, 95% CI: 0.99-1.07) for *L*_*den*_ with incidence of self-reported hypertension.^25^

More recent evidence on road traffic noise and factors related to hypertension has emerged since 2017, with outcome measures including antihypertensive,^26^^,^^27^ and treatment-resistant hypertension.^28^ The studies, conducted in Finland and Denmark, found a suggestive positive association with the antihypertensive. By contrast, the US study reported a significantly increased risk for treatment-resistant hypertension, with odds ratio per 10 dB[A] of 1.2 (95% CI: 1.0-1.4). There are 2 recent large studies that adopted an ecological design or used nationwide administrative data to explore the associations between road traffic noise and hypertension.^29^^,^^30^ Our findings are in line with the abovementioned 2 recent large studies. However, unlike our study, neither of these studies had data on lifestyle factors such as smoking status or alcohol intake at individual level. Another relatively small cohort study in Sweden (<5,000 participants) published in 2018 observed no association between road traffic noise exposure and incident hypertension.^31^ Despite variations among studies on the ascertainment or definition of hypertension, noise exposure assessment, and covariates adjusted for, our findings are supportive of a positive association between road traffic noise and incident hypertension.

To our knowledge, this is the first prospective study exploring the effects of road traffic noise exposure on the incidence of primary hypertension in the United Kingdom. Our previous cross-sectional study in UK Biobank has found that exposure to road traffic *L*_*den*_ >65 dB[A], as compared to ≤55 dB [A], was significantly associated with higher systolic blood pressure and diastolic blood pressure.^13^ This finding is supported by the current prospective analysis in which we found that long-term road traffic noise exposure was associated with a higher risk of incident primary hypertension in a dose-response relationship. A previous ecological study in London found no association between night-time road traffic noise and hypertension incidence with HRs of 1.00 (95% CI: 0.94-1.06) for >60 dB compared with <55 dB.^32^ That study, however, may be limited by the fact that the noise model applied was relatively too crude to detect small effects relating to nighttime noise exposure, for which the interquartile range across London boroughs was small (about 3 dB).

Our study found an increasing dose-response relationship between road traffic noise and incident primary hypertension when considered noise as a categorical variable, which is similar to the study in Toronto, Canada.^29^ A linear trend of exposure-response curves was found, which would provide supportive evidence of the exposure-response relationship of road traffic noise on incident primary hypertension.^33^

Given that road transportation results in both road traffic noise and air pollution, it is still incompletely understood as to whether exposures to each give additive or synergistic effects, or whether they may confound each other.^7^^,^^12^ A few studies have included both exposures when estimating associations with hypertension,^34^ and the results are somewhat variable. Our results showed that when noise was analyzed on a categorical scale, the significant associations found in the highest noise groups did not appear to be confounded by air pollution. Furthermore, the associations with road traffic noise on a continuous scale were also robust when adjusting for air pollution. The associations between air pollution (ie, PM_2.5_) and incident hypertension were independent of road traffic noise impacts in our study.

One important finding of our study is that road traffic noise and air pollution may have synergistic effects on the incidence of primary hypertension. Specifically, we observed strongest associations in participants who both exposed to both high traffic noise and traffic-related air pollutants PM_2.5_ or NO_2_ in the combined effects (*P* < 0.05).

It seems plausible that high levels of exposure to air pollution renders the body more sensitive to the hazardous effects of road traffic noise and vice versa. There are some potential mechanisms for the synergistic effects. Road traffic noise and air pollution are considered as triggers of hypertension,^35^ and they share some overlapping mechanisms, such as via vascular dysfunction, peripheral vasoconstriction, systemic inflammation, and hypothalamus-pituitary-adrenal axis activation, which may result in elevated blood pressure, and over the long-term lead to hypertension.^7^^,^^36^^,^^37^

Furthermore, it is concerning to find that the population who were residing in the most deprived areas had significantly higher risk of incident primary hypertension than their counterparts in the least deprived areas. Socioeconomic status could be a surrogate for environmental and nonenvironmental risk factors that have not been directly or fully measured, and change the physical environment in these regions might lead to greater absolute benefits and should be prioritized. Thus, the findings may suggest policy of targeted protection from traffic noise exposure in potentially susceptible populations.

Several notable strengths of the study are as follows: first, the longitudinal study design was performed on a very large sample size of more than 250,000 study participants across the United Kingdom with follow-up of more than 8 years and individual-level information on potential confounders. Second, an objectively-assessed diagnosis of primary hypertension was obtained by using linkage with medical records using ICD-10 (I10). Though the associations of long-term exposure to road traffic noise and incidence of hypertension are not fully consistent in prior studies,^25^^,^^29^^,^^32^^,^^34^^,^^38^ our findings added a high-quality evidence on this important issue.

### Study limitations

First, we only estimated noise exposure level at baseline address, without considering more recent long-term exposure prior to diagnosis of incident hypertension. Nevertheless, it is assumed that the residential noise level is stable during the follow-up period, since the building environment and design of road transportation and road surfaces change slowly over time.^39^ Second, road traffic noise levels were estimated from outdoor residential address rather than at individual level, where noise bears some uncertainty as time spent out of the residence, attenuation from the building structure, closing of windows, indoor noise level, etc. Such exposure misclassification may lead to the possible nondifferential exposure misclassification error and likely potential underestimation of our observed associations.^13^ In addition, we also examined *L*_*night*_, which may be inclined to reduce misclassification compared with *L*_*den*_, as most people stay at home during nighttime hours, and findings using *L*_*night*_ were similar to those of using *L*_*den*_. Third, although we adjusted for a number of potential confounders, residual confounding from unrecognized or unmeasured factors might be still present. Fourthly, our study population was limited to age 40 to 69 years—while this age group provides the majority of new primary hypertension diagnoses, the results may not be fully generalizable to other age groups. Finally, as the current study was conducted in the United Kingdom and most of the study participants were of European descent, results should be generalized to other populations with caution.

## Conclusions

In summary, within the context of a large prospective study of the middle-aged and elderly population in the UK Biobank, our results show that long-term residential exposure to road traffic noise is associated with elevated risk of incident primary hypertension. Furthermore, the effects are exacerbated by higher air pollution levels. Given the ubiquitous presence of road traffic noise and air pollution, these findings highlight the importance of minimizing exposure to road traffic noise and air pollution levels.

PERSPECTIVES **COMPETENCY IN MEDICAL KNOWLEDGE:** Given that currently nearly 3 million people in the United Kingdom are exposed to road traffic noise >65 dB, the roughly 13% increased risk of incident primary hypertension for *L*_*den*_ >65 dB[A] vs ≤55 dB[A] in our study has potentially important implications for the disease burden from hypertension in the United Kingdom. Furthermore, the effects appear to be exacerbated in higher air pollution levels.

**TRANSLATIONAL OUTLOOK:** Considering that all of the participants had air pollution exposures above the level of the 2021 WHO air quality guideline, also that the 2021 European Society of Cardiology Guideline on CVD included air pollution as well as above-threshold noise levels as modifiable environmental risk factors for CVD for the first time, our findings provide timely and important scientific evidence to inform clinical and public health policy and to reduce the disease burden of primary hypertension associated with road traffic noise and air pollution exposure.

## Funding support and author disclosures

This research was funded by the PEAK Urban programme, 10.13039/100014013UKRI’s Global Challenge Research Fund (grant no: ES/P011055/1). Dr Huang is funded by the State Scholarship Fund of 10.13039/501100004543China Scholarship Council (grant no: 202006015008). Dr Rahimi has received support from the National Institute for Health Research (NIHR) Oxford Biomedical Research Centre (BRC), the Oxford Martin school (OMS), University of Oxford and the British Heart Foundation (grant no: PG/18/65/33872). The views expressed are those of the authors and not necessarily those of the OMS, the UK National Health Service (NHS), the NIHR or the Department of Health and Social Care. Drs Gulliver, Hansell, and Cai have received support from the National Institute for Health Research (NIHR) Health Protection Research Unit (HPRU) in Environmental Exposures and Health at the University of Leicester development award, a partnership between the UK Health Security Agency, the Health and Safety Executive and the University of Leicester. The views expressed are those of the author(s) and not necessarily those of the NHS, the NIHR, the Department of Health and Social Care or the UK Health Security Agency. All other authors have reported that they have no relationships relevant to the contents of this paper to disclose.
