# Supplementary material for: Road Traffic Noise and Incidence of Primary Hypertension: A Prospective Analysis in UK Biobank
Source: JACC Adv. 2023 Mar 22;2(2):100262. doi: 10.1016/j.jacadv.2023.100262 (PMC10098371; doi:10.1016/j.jacadv.2023.100262)
Supplement: Supplementary Figure 1 and Tables 1–5 [file mmc1.docx]

**Supplemental Table 1.** The Spearman correlations between weight average 24-h road traffic noise (*L_den_*), night-time road traffic noise (*L_night_*), and air pollution

| **Variables** | ***L_den_*** | ***L_night_*** | **PM_2.5_** | **NO_2_** |
| --- | --- | --- | --- | --- |
| *L_den_* | 1.00 |  |  |  |
| *L_night_* | 1.00 | 1.00 |  |  |
| PM_2.5_ | 0.24 | 0.24 | 1.00 |  |
| NO_2_ | 0.23 | 0.23 | 0.86 | 1.00 |

*L_den_*: weighted average 24-h road traffic noise level;

*L_night_* : average night-time road traffic noise level from 23:00 to 7:00;

PM_2.5_, fine particles; NO_2_, nitrogen dioxide.

**Supplemental Table 2**. Combined effects of night-time road traffic noise (*L_night_*) and air pollution in relation to risk of incidence of primary hypertension

| **Air pollution** | | **The influence of night road traffic noise exposure (*L_night_*)** | | | | |
| --- | --- | --- | --- | --- | --- | --- |
|  |  | ***Low***  **(≤45 dB[A])** | ***Low-medium***  **(>45 to ≤50 dB[A])** | ***Medium-high***  **(>50 to ≤55 dB[A])** | | ***High***  **(>55 dB[A])** |
| PM_2.5_ | ≤9.9 μg/m^3^ | ref | 1.01 (0.94, 1.08) | 1.08 (0.94, 1.25) | 1.00 (0.84, 1.19) | |
|  | 9.9～10.6 μg/m^3^ | 0.99 (0.91, 1.08) | 1.02 (0.94, 1.11) | 0.98 (0.80, 1.21) | **1.26 (1.02, 1.55)** | |
|  | >10.6 μg/m^3^ | 1.06 (0.95, 1.18) | 1.05 (0.96, 1.14) | 1.08 (0.93, 1.26) | **1.22 (1.06, 1.40)** | |
| NO_2_ | ≤26.0 μg/m^3^ | ref | 1.02 (0.96, 1.09) | 1.05 (0.93, 1.19) | 0.98 (0.82, 1.18) | |
|  | 26.0-31.1 μg/m^3^ | 1.02 (0.94, 1.11) | 1.07 (0.99, 1.16) | 1.07 (0.90, 1.29) | 1.21 (0.98, 1.49) | |
|  | >31.1 μg/m^3^ | 1.01 (0.91, 1.13) | 0.96 (0.88, 1.05) | 1.14 (0.97, 1.35) | **1.19 (1.05, 1.35)** | |

PM_2.5_ and NO_2_ concentrations were divided by ≤*P_50_*, *P_50_* and *P_75_* , and > *P_75_.*

PM_2.5_, fine particles; NO_2_, nitrogen dioxide. Bold represents significance at *P* < 0.05.

**Supplemental Table 3.** The associations between exposure to weight average 24-h road traffic noise (*L_den_*) and incidence of primary hypertension stratified by personal characteristics

| **Variables** | **Low**  **(≤55 dB[A])** | | **Low-medium**  **(>55 to ≤60 dB[A])** | | **Medium-high**  **(>60 to ≤65 dB[A])** | | **High**  **(>65 dB[A])** | |
| --- | --- | --- | --- | --- | --- | --- | --- | --- |
|  | **HR (95%CI)** | ***P_interaction_*** | **HR (95%CI)** | ***P_interaction_*** | **HR (95%CI)** | ***P_interaction_*** | **HR (95%CI)** | ***P_interaction_*** |
| **Age** |  |  |  |  |  |  |  |  |
| <65 years | ref | ref | 0.97 (0.94, 1.01) | ref | 1.01 (0.94, 1.09) | ref | 1.03 (0.96, 1.11) | ref |
| ≥65 years | ref | ref | 1.02 (0.95, 1.09) | 0.308 | 0.96 (0.84, 1.10) | 0.350 | 1.07 (0.94, 1.22) | 0.546 |
| **Sex** |  |  |  |  |  |  |  |  |
| Female | ref | ref | 0.97 (0.91, 1.04) | ref | 1.12 (0.98, 1.30) | ref | 1.07 (0.93, 1.24) | ref |
| Male | ref | ref | **1.07 (1.00, 1.14)** | 0.068 | 1.00 (0.88, 1.14) | 0.652 | **1.19 (1.04, 1.35)** | 0.613 |
| **Economic status** | |  |  |  |  |  |  |  |
| Inactive | ref | ref | 1.03 (0.96, 1.10) | ref | 1.04 (0.91, 1.19) | ref | **1.17 (1.01, 1.34)** | ref |
| Active | ref | ref | 1.02 (0.96, 1.10) | 0.957 | 1.07 (0.93, 1.22) | 0.771 | 1.11 (0.97, 1.27) | 0.839 |
| **Townsend deprivation index** | |  |  |  |  |  |  |  |
| 1 (least deprived) | ref | ref | 0.94 (0.81, 1.10) | ref | 1.07 (0.77, 1.49) | ref | 0.94 (0.66, 1.34) | ref |
| 2 | ref | ref | 1.13 (0.96, 1.33) | 0.065 | 1.10 (0.80, 1.53) | 0.913 | 1.19 (0.81, 1.73) | 0.076 |
| 3 | ref | ref | 0.99 (0.84, 1.17) | 0.638 | 1.03 (0.72, 1.47) | 0.932 | 1.16 (0.83, 1.62) | 0.243 |
| 4 | ref | ref | 1.06 (0.89, 1.28) | 0.344 | 1.01 (0.70, 1.45) | 0.787 | 1.31 (0.94, 1.83) | 0.051 |
| 5 (most deprived) | ref | ref | 1.06 (0.87, 1.28) | 0.086 | 0.95 (0.68, 1.33) | 0.769 | 1.31 (0.94, 1.82) | 0.020 |
| **Length of time at residence** | |  |  |  |  |  |  |  |
| <10 years | ref | ref | 1.08 (0.96, 1.20) | ref | 1.08 (0.87, 1.33) | ref | **1.25 (1.01, 1.53)** | ref |
| ≥10 years | ref | ref | 1.01 (0.96, 1.07) | 0.728 | 1.05 (0.94, 1.17) | 0.456 | 1.11 (0.99, 1.23) | 0.731 |
| **Salt intake** | |  |  |  |  |  |  |  |
| Never/rarely | ref | ref | 1.00 (0.93, 1.08) | ref | **1.19 (1.02, 1.39)** | ref | 1.15 (0.98, 1.35) | ref |
| Sometimes | ref | ref | 0.99 (0.87, 1.12) | 0.109 | 0.85 (0.66, 1.11) | 0.183 | 1.07 (0.82, 1.40) | 0.244 |
| Usually | ref | ref | 1.14 (0.87, 1.49) | 0.234 | 1.37 (0.83, 2.23) | 0.834 | 1.15 (0.69, 1.92) | 0.535 |
| Always | ref | ref | 0.52 (0.21, 1.27) | 0.518 | 0.91 (0.14, 5.87) | 0.843 | 0.39 (0.04, 3.63) | 0.725 |
| **Sedentary time** | |  |  |  |  |  |  |  |
| ≤4.5 hours | ref | ref | 0.98 (0.91, 1.06) | ref | 0.96 (0.82, 1.12) | ref | 1.12 (0.96, 1.31) | ref |
| >4.5 hours | ref | ref | 1.06 (0.98, 1.16) | 0.153 | 1.10 (0.92, 1.30) | 0.193 | **1.33 (1.12, 1.59)** | 0.614 |
| **Physical activity level** | |  |  |  |  |  |  |  |
| Low | ref | ref | 0.96 (0.76, 1.21) | ref | 0.79 (0.48, 1.29) | ref | 1.25 (0.75, 2.07) | ref |
| Moderate | ref | ref | 1.00 (0.90, 1.11) | 0.480 | 0.89 (0.72, 1.09) | 0.071 | 1.07 (0.85, 1.34) | 0.958 |
| High | ref | ref | 1.05 (0.94, 1.17) | 0.991 | 1.18 (0.94, 1.47) | 0.432 | 1.16 (0.93, 1.45) | 0.433 |

Bold represents significance at *P* < 0.05.

**Supplemental Table 4.** The association between exposure to night-time road traffic noise (*L_night_*) and incidence of first occurrence of primary hypertension stratified by personal characteristics

| **Variables** | **Low**  **(≤45 dB[A])** | | | **Low-medium**  **(>45 to ≤50 dB[A])** | | | | **Medium-high**  **(>50 to ≤55 dB[A])** | | | **High**  **(>55 dB[A])** | | | |
| --- | --- | --- | --- | --- | --- | --- | --- | --- | --- | --- | --- | --- | --- | --- |
|  | **HR (95%CI)** | ***P_interaction_*** | | **HR (95%CI)** | | ***P_interaction_*** | | **HR (95%CI)** | ***P_interaction_*** | | **HR (95%CI)** | | ***P_interaction_*** | |
| **Age** |  |  | |  | |  | |  |  | |  | |  | |
| <65 years | ref | ref | | 0.98 (0.94, 1.01) | | ref | | 0.99 (0.92, 1.06) | ref | | 1.03 (0.96, 1.11) | | ref | |
| ≥65 years | ref | ref | | 1.01 (0.95, 1.08) | | 0.365 | | 1.01 (0.89, 1.15) | 0.981 | | 1.08 (0.95, 1.24) | | 0.550 | |
| **Sex** |  |  | |  | |  | |  |  | |  | |  | |
| Female | ref | ref | | 0.98 (0.92, 1.06) | | ref | | 1.12 (0.98, 1.28) | ref | | 1.09 (0.95, 1.25) | | ref | |
| Male | ref | ref | | 1.03 (0.97, 1.10) | | 0.164 | | 1.04 (0.92, 1.17) | 0.564 | | **1.16 (1.03, 1.32)** | | 0.821 | |
| **Economic status** | |  |  | |  | |  | |  |  | |  | |  |
| Inactive | ref | ref | | 1.02 (0.95, 1.09) | | ref | | 1.07 (0.94, 1.21) | ref | | **1.15 (1.01, 1.32)** | | ref | |
| Active | ref | ref | | 1.01 (0.94, 1.08) | | 0.479 | | 1.08 (0.95, 1.23) | 0.963 | | 1.11 (0.98, 1.27) | | 0.890 | |
| **Townsend deprivation index** | |  |  | |  | |  | |  |  | |  | |  |
| 1 (least deprived) | ref | ref | | 0.91 (0.78, 1.05) | | ref | | 1.06 (0.78, 1.45) | ref | | 0.91 (0.65, 1.29) | | ref | |
| 2 | ref | ref | | 1.16 (0.99, 1.36) | | 0.150 | | 1.12 (0.82, 1.53) | 0.904 | | 1.16 (0.81, 1.67) | | 0.072 | |
| 3 | ref | ref | | 1.02 (0.86, 1.21) | | 0.979 | | 1.01 (0.73, 1.41) | 0.883 | | 1.17 (0.84, 1.64) | | 0.226 | |
| 4 | ref | ref | | 0.99 (0.82, 1.20) | | 0.822 | | 1.02 (0.73, 1.44) | 0.798 | | 1.26 (0.90, 1.75) | | 0.092 | |
| 5 (most deprived) | ref | ref | | 1.08 (0.88, 1.32) | | 0.057 | | 1.10 (0.80, 1.53) | 0.858 | | 1.33 (0.96, 1.85) | | 0.006 | |
| **Length of time at residence** | |  |  | |  | |  | |  |  | |  | |  |
| <10 years | ref | ref | | 1.01 (0.90, 1.13) | | ref | | 1.03 (0.84, 1.26) | ref | | **1.23 (1.00, 1.50)** | | ref | |
| ≥10 years | ref | ref | | 1.01 (0.96, 1.07) | | 0.782 | | 1.08 (0.98, 1.20) | 0.099 | | 1.10 (0.99, 1.23) | | 0.811 | |
| **Salt intake** | |  |  | |  | |  | |  |  | |  | |  |
| Never/rarely | ref | ref | | 1.00 (0.93, 1.08) | | ref | | **1.18 (1.02, 1.37)** | ref | | 1.16 (0.99, 1.35) | | ref | |
| Sometimes | ref | ref | | 0.98 (0.86, 1.12) | | 0.960 | | 0.88 (0.69, 1.12) | 0.328 | | 1.13 (0.87, 1.47) | | 0.371 | |
| Usually | ref | ref | | 1.04 (0.80, 1.36) | | 0.889 | | 1.11 (0.68, 1.80) | 0.559 | | 1.14 (0.68, 1.90) | | 0.796 | |
| Always | ref | ref | | 0.62 (0.26, 1.50) | | 0.874 | | 0.33 (0.06, 1.95) | 0.769 | | 0.46 (0.05, 4.24) | | 0.599 | |
| **Sedentary time** |  |  | |  | |  | |  |  | |  | |  | |
| ≤4.5 hours | ref | ref | | 0.99 (0.92, 1.07) | | ref | | 1.02 (0.88, 1.18) | ref | | 1.10 (0.94, 1.28) | | ref | |
| >4.5 hours | ref | ref | | 1.04 (0.96, 1.13) | | 0.490 | | 1.07 (0.91, 1.26) | 0.632 | | **1.33 (1.12, 1.58)** | | 0.363 | |
| **Physical activity level** | |  |  | |  | |  | |  |  | |  | |  |
| Low | ref | ref | | 1.01 (0.80, 1.28) | | ref | | 0.80 (0.51, 1.26) | ref | | 1.17 (0.71, 1.93) | | ref | |
| Moderate | ref | ref | | 0.99 (0.89, 1.10) | | 0.340 | | 0.91 (0.74, 1.11) | 0.026 | | 1.06 (0.85, 1.33) | | 0.839 | |
| High | ref | ref | | 1.03 (0.92, 1.15) | | 0.764 | | 1.20 (0.97, 1.48) | 0.381 | | 1.14 (0.91, 1.42) | | 0.381 | |

Bold represents significance at *P* < 0.05.

**Supplemental Table 5.** Sensitivity analysis of estimated associations between exposure to road traffic noise (*L_den_* and *L_night_*) and incidence of primary hypertension

|  | **Model 1,**  **HR (95% CI)** | **Model 2,**  **HR (95% CI)** | **Model 3,**  **HR (95% CI)** |
| --- | --- | --- | --- |
| *L_den_* |  |  |  |
| Low (≤55 dB[A]) | ref | ref | ref |
| Low-medium (>55 to ≤60 dB[A]) | 1.00 (0.95, 1.06) | 1.03 (0.98, 1.08) | 1.02 (0.97, 1.07) |
| Medium-high (>60 to ≤65 dB[A]) | 1.05 (0.94, 1.17) | 1.06 (0.96, 1.17) | 1.06 (0.96, 1.18) |
| High (>65 dB[A]) | **1.17 (1.05, 1.31)** | **1.14 (1.04, 1.26)** | **1.14 (1.03, 1.27)** |
| *P* for trend | 0.006 | 0.004 | 0.009 |
| Continuous (per 10 dB[A] increase) | **1.09 (1.02, 1.16)** | **1.08 (1.03, 1.14)** | **1.07 (1.02, 1.13)** |
| *L_night_* |  |  |  |
| Low (≤45 dB[A]) | ref | ref | ref |
| Low-medium (>45 to ≤50 dB[A]) | 0.99 (0.94, 1.05) | 1.02 (0.97, 1.07) | 1.00 (0.95, 1.06) |
| Medium-high (>50 to ≤55 dB[A]) | 1.07 (0.96, 1.18) | 1.09 (0.99, 1.19) | 1.09 (0.99, 1.21) |
| High (>55 dB[A]) | **1.17 (1.05, 1.30)** | **1.14 (1.04, 1.26)** | **1.14 (1.02, 1.26)** |
| *P* for trend | 0.003 | 0.002 | 0.005 |
| Continuous (per 10 dB[A] increase) | **1.09 (1.02, 1.16)** | **1.08 (1.03, 1.14)** | **1.07 (1.02, 1.13)** |

Model 1: fully adjusted model plus adjusted for central obesity status and physical activity level.

Model 2: fully adjusted model plus adjusted for sedentary time and sleep duration.

Model 3: exclude those who were identified primary hypertension within two year during the follow-up period based on the fully adjusted model.

Bold represents significance at *P* < 0.05.

**Supplemental Figure 1.** Directed Acyclic Graph displaying the relationship between road traffic noise, hypertension and other variables
